# Substantial variation across geographic regions in the obesity prevalence among 6–8 years old Hungarian children (COSI Hungary 2016)

**DOI:** 10.1186/s12889-018-5530-6

**Published:** 2018-05-09

**Authors:** Gergő Erdei, Márta Bakacs, Éva Illés, Barbara Nagy, Csilla Kaposvári, Erzsébet Mák, Eszter Sarkadi Nagy, Zoltán Cserháti, Viktória Anna Kovács

**Affiliations:** 1 School of PhD Studies, Doctoral School of Pathological Sciences, Health Science Research, Semmelweis University, 26 Üllői Street, Budapest, 1085 Hungary; 2 Division of Nutrition, National Institute of Pharmacy and Nutrition, 3 Zrínyi Street, Budapest, 1051 Hungary; 3 Department of Dietetics and Nutrition Sciences, Faculty of Health Sciences, Semmelweis University, 17 Vas Street, Budapest, 1088 Hungary

**Keywords:** Overweight, Obesity, Prevalence, Children, Hungary, COSI, Urbanization, Geographic regions

## Abstract

**Background:**

There have been previous representative nutritional status surveys conducted in Hungary, but this is the first one that examines overweight and obesity prevalence according to the level of urbanization and in different geographic regions among 6–8-year-old children. We also assessed whether these variations were different by sex.

**Methods:**

This survey was part of the fourth data collection round of World Health Organization (WHO) Childhood Obesity Surveillance Initiative which took place during the academic year 2016/2017. The representative sample was determined by two-stage cluster sampling. A total of 5332 children (48.4% boys; age 7.54 ± 0.64 years) were measured from all seven geographic regions including urban (at least 500 inhabitants per square kilometer; *n* = 1598), semi-urban (100 to 500 inhabitants per square kilometer; *n* = 1932) and rural (less than 100 inhabitants per square kilometer; *n* = 1802) areas.

**Results:**

Using the WHO reference, prevalence of overweight and obesity within the whole sample were 14.2, and 12.7%, respectively. According to the International Obesity Task Force (IOTF) reference, rates were 12.6 and 8.6%. Northern Hungary and Southern Transdanubia were the regions with the highest obesity prevalence of 11.0 and 12.0%, while Central Hungary was the one with the lowest obesity rate (6.1%). The prevalence of overweight and obesity tended to be higher in rural areas (13.0 and 9.8%) than in urban areas (11.9 and 7.0%). Concerning differences in sex, girls had higher obesity risk in rural areas (OR = 2.0) but boys did not. Odds ratios were 2.0–3.4 in different regions for obesity compared to Central Hungary, but only among boys.

**Conclusions:**

Overweight and obesity are emerging problems in Hungary. Remarkable differences were observed in the prevalence of obesity by geographic regions. These variations can only be partly explained by geographic characteristics.

**Trial registration:**

Study protocol was approved by the Scientific and Research Ethics Committee of the Medical Research Council (61158–2/2016/EKU).

## Background

Overweight and obesity among children are one of the most important public health issues of our time [1]. Child and adolescent overweight has significant medical and non-medical impacts both in childhood and later in life [2]. Approximately 7% of national health budgets are spent on diseases linked to obesity each year in the EU [3]. Therefore, childhood obesity is a particular challenge for a health system with limited resources such as the one in Hungary [4].

Several studies investigated the differences in obesity prevalence between urban and rural areas, but results are controversial. The majority of studies found higher overweight and obesity prevalence in rural areas compared to urban areas [5–7], however others have found the opposite [7, 8]. Besides, the pathways that lead to differences in the prevalence between urban and rural areas are not well understood. Possible explanations might be the social and cultural differences which in some cases are influenced by lower educational attainment, and the variety in the presence of certain risk factors between urban and rural environments such as less possibilities for physical activity due to higher distance to recreational facilities, transport options or due to safety concerns for active mobility in rural areas [9–12]. More possibilities for eating out particularly in fast food restaurant or higher costs of fruit and vegetables in urban settlements may also explain partly this phenomena [13]. Finally, residents in rural areas may lack access to primary prevention efforts [14].

Research investigating regional differences in obesity prevalence have received less attention, particularly in child population. It appears that area level socio-economic and cultural factors are important predictors of childhood obesity [15], however the exact causes of regional differences in the prevalence rates are not clear yet [16].

Given that the social [17], cultural [18] and economic context [19] as well as the built environment [20] seem to have great impact on weight status, and the fact that these factors differ both between rural versus urban areas and by regions, this paper aimed to study the overweight and obesity prevalence both according to urbanization level and in different geographic regions. The second aim was to get information about the sex effect on these differences. Our hypothesis was that there are differences in overweight and obesity prevalence among Hungarian children both by regions and by urbanization levels, and sex has an impact on these varieties.

## Methods

Childhood Obesity Surveillance Initiative (COSI) was initiated by the WHO Regional Office for Europe in 2007 [21]. According to COSI protocol the participant countries collect measured data on the prevalence of overweight and obesity using standardized methods applying on nationally representative samples of children aged 6–9 years [22]. COSI is a repeated cross-sectional study, which ─ besides the anthropometric measurements ─ collects data about school environment (mandatory part) and about dietary and activity habits on individual level (voluntary part).

The COSI protocol is in accordance with the International Ethical Guidelines for Biomedical Research Involving Human Subjects [23, 24]. The Hungarian COSI survey was approved by the National Scientific and Ethical Committee (61158–2/2016/EKU).

### Sampling design

Target population for Hungary was defined as children aged 7.0–7.9 years on September 1, 2016. Following the COSI protocol, we aimed for a minimum sample size of 2800 pupils. Assuming 90% response rate and taking a design effect of 1.2 we have planned to enroll 3100 children. This design at 80% power allowed us to detect a minimum difference of 0.10 Z-score in mean BMI per year at a two-sided 5% significance level. To draw a national representative sample, two-stage stratified sampling procedure was applied. The first stage sampling was stratified on counties. In this stage 155 schools from the sampling frame of size 2370 were selected. For the second stage we used a simple random sampling, choosing one 1st and 2nd classes within each previously selected first stage. Schools which had merged classes with less than 10 students in the 1st grade or did not have 1st grade classes (*N* = 593) were excluded. Schools for children with special needs (*N* = 75) and schools owned by private persons or companies (*N* = 12) were also excluded. The final sample comprised of 155 schools with 310 1st and 2nd grade classes.

The final data set contained 5454 measured children of which 16 were excluded (height was out of the range of mean ± 3SD). For the sake of comparability, exclusion criteria was the same as in the previous COSI Hungary round. Although the targeted population was the 7-year-old children (*N* = 2651), participation of 6-year-olds (*N* = 1180) and 8-year-olds (*N* = 1501) were significant, thus, this paper also includes these data in the analysis (Table 1).

**Table 1:** Descriptive characteristics of study participants

	Number	Percent
Total Girls Boys	5332 2579 2753	100 51.6 48.4
Age		
6-year-old 7-year-old 8-year-old	1180 2651 1501	22.1 49.7 28.2
Geographic region		
Central Hungary Western Transdanubia Central Transdanubia Southern Transdanubia Northern Hungary Northern Great Plain Southern Great Plain	1524 491 539 386 670 970 752	28.6 9.2 10.1 7.2 12.6 18.2 14.1
Level of urbanization^a^		
Urban Semi-urban Rural	1598 1932 1802	30.0 36.2 33.8

^a^Urban: ≥ 500 inhabitants/ km2. Semi-urban: < 500 and ≤ 100 inhabitants/ km2. Rural: < 100 inhabitants/ km2

### Study procedures

In 2016, only the mandatory COSI elements were carried out in Hungary. These are the anthropometric measurements (body height and body weight) and the COSI mandatory school record form collecting information about the school environment. Data were gathered in a 4-week-long period between 3rd and 31st October, 2016. An opt-out consent approach was used, so if the parents did not want their children to participate in the study, they would get in touch with the research team.

Anthropometric measurements were carried out by 134 school nurses. Body weight and height were measured according to WHO standardized techniques. All fieldworkers received a training CD about measuring tools, assembly guidelines and a demonstration video on the height and weight measurements using WHO standards [23]. Before the measurements, school nurses recorded the following data on individual level: date of birth, sex, place of living (only the name of the city, town or village), clothes worn at the time of measurement and whether breakfast was consumed on that day. Time of measurements was also noted. Children’s verbal permission was requested before taking the measurements. Children were asked to remove their shoes as well as heavy clothing (sweaters, jackets etc.) and other personal items (wallets, mobile phones, key chains etc.). Most children were dressed in gym clothes (48.2%) or in light clothing (40.2%). Body weight was measured to the nearest 0.1 kg (kg) using portable digital OMRON BF511 weight scales, and body height was measured standing upright, to the nearest 0.1 cm (cm) using 2 M wall mounted stadiometer (model number: ar6547) roll-up height measurer. Weight and height measurements were taken only once for each child.

A member of the expert team from the national coordinating institute verified the completeness of forms, schools with incomplete questionnaires were contacted and the missing responses were supplemented.

### Definition of overweight and obesity

To ensure comparability with other works, prevalence rates are presented both according to the IOTF [25, 26] (overweight was defined as age-and-sex specific ≥25 and < 30 BMI, and obesity was defined as age-and-sex specific ≥30 BMI) and to the WHO (overweight was defined as age-and-sex specific > 1 Standard Deviation (SD) and ≤ 2 SD, and obesity was defined as age-and-sex specific > 2 SD) cut-off points [27, 28]. However, for analyzing the differences by the level of urbanization and by regions prevalence rates are only demonstrated according to the IOTF criteria as these cut-off values are closer to Hungarian national cut-off values [29] than the WHO cut-offs, particularly for obesity where the WHO cut-offs are much lower than either the national or the IOTF cut-offs [30].

### Geographic location and urbanization grade

**Fig. 1:**
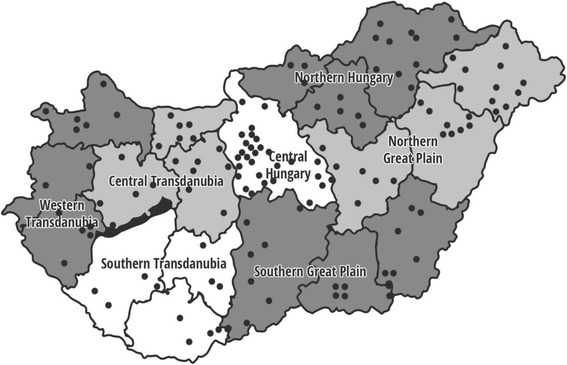
Geographic distribution of the 136 primary schools participated in 2016 to be representative for Hungary. Each dot represents one school. Source of map: https://pixabay.com/hu/megye-magyarorsz%C3%A1g-t%C3%A9rk%C3%A9p-vector-892470/. Dots representing participating schools were added by the first author of the manuscript

Hungary can be divided into seven regions: Northern Hungary, Northern Great Plain, Southern Great Plain, Southern Transdanubia, Western Transdanubia, Central Transdanubia, and Central Hungary (Fig. 1). Beyond the geographic distribution, residence of children were grouped into urban, semi-urban or rural categories as follows: a) urban: the population density is at least 500 inhabitants per square kilometer; b) semi-urban: between 100 and 500 inhabitants per square kilometer; c) rural: the population density is less than 100 inhabitants per square kilometer [31].

### Data processing

All data was processed anonymously. EpiData Entry 3.1 software was used for data entry, which included built-in range (e.g. outliers, out of range values) and consistency checks for validation. Two independent data clerks entered the individual data two times to provide quality assurance. In order to get the exact age of each child, the birthdate was subtracted from the measurement date then variables with age in years were created. The exact weight of each child was adjusted for the weight of clothes worn (− 0.13 kg for gym clothes, − 0.195 kg for light clothing and − 0.6 kg for heavy clothing) then body mass index (BMI, kg/m2) was calculated.

Using the survey data, we performed descriptive statistical analysis and built regression model to assess association between prevalence of overweight and obesity, and a few health determinants. By applying sampling weight developed according to the sampling procedure, the analysis was fitted to sampling characteristics. For each country a weight was calculated based on the proportion of population groups formed by sex and age. Taking into account the two-stage sampling procedure, we used the school-ID for calculating the final design weight. Pearson χ^2^ test was used for categorical variables to test gender differences. The mean values between two independent samples were compared using independent sample t-test after testing for normality. Multivariable logistic regression was used to estimate odds-ratios (OR) for childhood overweight and obesity with 95% confidence interval (95% CI). For dependent variable overweight or obesity was used; level of urbanization, age, and different geographical regions as independent variables at once were included in the models. A *p* value < 0.05 level was considered statistically significant. We used STATA 11 statistical software for population estimates and data analysis.

## Results

The current population estimates are based on the representative sample of 5332 schoolchildren aged 6.0 to 8.99 years (mean age 7.54 ± 0.64 years; 48.4% boys). Table 1 summarizes participants’ characteristics. Concerning the level of urbanization, number of children in the urban, semi-urban and rural were approximately equal.

Table 2 presents the anthropometric variables in the studied population. Height and weight rise with age in both sex. Boys in every age group are taller and heavier than girls, but significant sex difference was only obtained in height among 7-year-olds (126.6 cm vs. 127.7 cm; *p* < 0.0001) and in weight among 8-year-old children (28.7 kg vs. 29.9 kg; *p* < 0.003). BMI rose parallel with age except in girls between 7 and 8 years (16.7 kg/m^2^ vs. 16.7 kg/m^2^). We found relevant but statistically not significant BMI difference between 8-year-old boys and girls (17.1 kg/m^2^ vs. 16.7 kg/m^2^).

**Table 2:** Anthropometric variables in 6–8-year-old Hungarian schoolchildren (based on population estimates)

		Total	Girls	Boys	Sex difference *p*-value*
	Age	Mean (95% CI)	Mean (95% CI)	Mean (95% CI)	
Height (cm)	6-year-old	122.8 (122.3; 123.2)	122.4 (121.9; 122.9)	123.1 (122.4; 123.7)	0.066
	7-year-old	127.1 (126.8; 127.4)	126.6 (126.2; 126.9)	127.7 (127.2; 128.1)	< 0.0001
	8-year-old	131.2 (130.8; 131.6)	130.5 (129.9; 131.1)	131.9 (131.4; 132.3)	0.760
Weight (kg)	6-year-old	24.5 (24.2; 24.9)	24.3 (23.9; 24.7)	24.7 (24.3; 25.2)	0.096
	7-year-old	27.1 (26.8; 27.3)	26.9 (26.6; 27.3)	27.2 (26.9; 27.6)	0.241
	8-year-old	29.3 (28.9; 29.7)	28.7 (28.1; 29.3)	29.9 (29.4; 30.5)	0.003
BMI (kg/m^2^)	6-year-old	16.2 (16.0; 16.4)	16.1 (15.9; 16.3)	16.2 (16.0; 16.5)	0.351
	7-year-old	16.6 (16.5; 16.8)	16.7 (16.5; 16.9)	16.6 (16.4; 16.8)	0.442
	8-year-old	16.9 (16.7; 17.1)	16.7 (16.5; 17.0)	17.1 (16.8; 17.3)	0.061

*BMI* body mass index, *CI* confidence interval

* *p*-values (continuous variables) are calculated with t test

Significant *p*-value: *p* < 0.05

Prevalence of overweight and obesity are described in Table 3. Depending on which definition we used the prevalence of overweight varied from 13.1 to 16.4% in girls and from 9.6 to 15.3% in boys. The prevalence of obesity was 7.4–12.1% in girls and 8.2–16.0% in boys. We could not detect significant sex difference in overweight and obesity prevalence among 6–8-year-olds, except for obesity defined using WHO criteria. The pattern in boys and girls were different: overweight and obesity rates increased with age among boys but not in girls where the highest values were seen among the 7-year-olds.

**Table 3:** Prevalence of overweight and obesity among 6–8-year-old Hungarian schoolchildren (based on population estimates)

		IOTF/Cole^a^		WHO 2007^b^	
		Overweight	Obesity	Overweight	Obesity
Total	6 y % (95% CI)	11.3 (9.5;13.5)	8.3 (6.5; 10.4)	12.6 (10.8; 14.7)	11.3 (9.3; 13.7)
	7 y % (95% CI)	13 (11.7; 14.5)	9.5 (8.3; 10.7)	14.8 (13.5; 16.3)	13.6 (12.1; 15.2)
	8 y % (95% CI)	13.4 (11.8; 15.2)	8.2 (6.9; 9.6)	15.0 (12.9–17.3)	13.0 (11.3; 14.9)
Girls	6 y % (95% CI)	13.1 (10.5; 16.4)	8.3 (6.2; 10.8)	13.3 (10.6; 16.5)	10.8 (8.5; 13.6)
	7 y % (95% CI)	13.6 (11.6; 15.8)	10.1 (8.4; 12.0)	16.4 (14.5; 18.5)	12.1 (10.3; 14.3)
	8 y % (95% CI)	13.2 (10.4; 16.5)	7.4 (5.7; 9.5)	14.7 (11.7; 18.4)	9.9 (7.8; 12.4)
Boys	6 y % (95% CI)	9.6 (7.4–12.4)	8.2 (5.8–11.5)	12.1 (9.6; 15.0)	11.8 (9.0; 15.3)
	7 y % (95% CI)	12.5 (10.8; 14.5)	8.9 (7.5; 10.5)	13.3 (11.3; 15.5)	15.0 (13.1; 17.2)
	8 y % (95% CI)	13.6 (11.5–15.9)	8.9 (7.1–11.3)	15.3 (12.8; 18.1)	16.0 (13.4; 19.0)
Sex difference *p*-value*		0.1624	0.8957	0.2240	0.0011

*CI* confidence interval

*p values (categorical variables) are calculated with Pearson χ^2^ test to compare the combined prevalence (age 6–8 years old) of overweight and obesity between boys and girls

^a^ Based on IOTF reference [25, 26]

^b^ Based on the WHO growth reference [27]

**Fig. 2:**
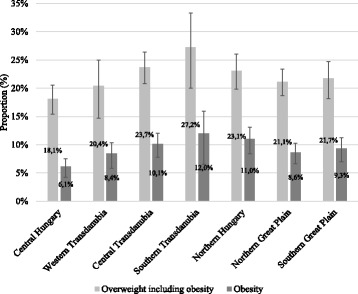
Weight classification of 6–8 years old schoolchildren by geographic regions, 95% CI. Based on IOTF reference [25, 26]. CI = confidence interval

**Fig. 3:**
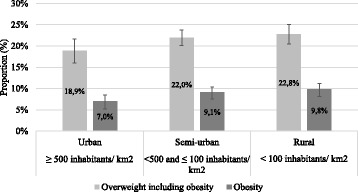
Weight classification of 6–8 years old schoolchildren by the level of urbanization, 95% CI. Based on IOTF reference [25, 26]. CI = confidence interval

Figures 2 and 3 present the prevalence rates according to the level of urbanization and in the different geographic regions. We obtained significant differences in the prevalence rates of overweight and obesity among the seven geographic regions (*p* = 0.0402). Both overweight (including obesity) and obesity were most frequent in Southern Transdanubia (27.2%; 12.0%) while the lowest rates were found in Central Hungary (18.1%; 6.1%) and in Western Transdanubia (20.4%; 8.4%). The obesity prevalence in the region with the highest rate was two times higher than in the one with the lowest rate (12% vs. 6.1%). Overweight and obesity were more common in rural than in urban regions but these variations were not significant.

The multivariable analysis showed distinct results by sex (Table 4).

**Table 4:** Odds ratio for overweight and obesity in 6–8 years old Hungarian girls and boys

	Girls				Boys			
	Overweight^¤^		Obesity^¤^		Overweight^¤^		Obesity^¤^	
	OR (95% CI)	P-value	OR (95% CI)	P-value	OR (95% CI)	P-value	OR (95% CI)	P-value
Age								
6-year-old	1.0		1.0		1.0		1.0	
7-year-old	1.0 (0.7; 1.4)	0.863	1.2 (0.9; 1.8)	0.240	1.3 (1; 1.8)	0.067	1.1 (0.7; 1.6)	0.652
8-year-old	1.0 (0.7; 1.5)	0.973	0.9 (0.6; 1.3)	0.533	1.5 (1.1; 2.0)	0.021	1.1 (0.7; 1.7)	0.688
Geographic region								
Central Hungary	1.0		1.0		1.0		1.0	
Western Transdanubia	1.5 (1.0; 2.2)	0.076	1.2 (0.7; 2.0)	0.555	0.8 (0.5; 1.3)	0.410	2.1 (1.1; 4.1)	0.032
Central Transdanubia	0.9 (0.5; 1.5)	0.684	0.7 (0.4; 1.2)	0.211	1.1 (0.7; 1.7)	0.729	2.3 (1.2; 4.2)	0.009
Southern Transdanubia	1.5 (0.8; 2.8)	0.233	1.0 (0.5; 2.1)	0.996	1.1 (0.6; 2.0)	0.690	3.4 (1.6; 7.3)	0.002
Northern Hungary	1.0 (0.7; 1.5)	0.840	1.3 (0.8; 2.2)	0.286	0.9 (0.6; 1.6)	0.816	2.4 (1.4; 4.1)	0.001
Northern Great Plain	1.0 (0.7; 1.6)	0.830	0.8 (0.5; 1.2)	0.263	1.0 (0.6; 1.6)	0.899	2.2 (1.2; 4.1)	0.012
Southern Great Plain	1.2 (0.8; 1.8)	0.430	1.0 (0.6; 1.7)	0.958	0.9 (0.5; 1.3)	0.477	2.0 (1.1; 3.5)	0.021
Level of urbanization								
Urban	1.0		1.0		1.0		1.0	
Semi-urban	1.1 (0.8; 1.5)	0.566	1.4 (1.0; 2.2)	0.079	1.0 (0.7; 1.4)	0.952	0.9 (0.6; 1.5)	0.667
Rural	1.0 (0.7; 1.4)	0.938	2.0 (1.3; 3.1)	0.003	1.1 (0.7; 1.7)	0.670	0.7 (0.4; 1.2)	0.182

Based on IOTF reference [25, 26]

Adjusted for age, geographic region and level of urbanization

Significant at p-value *p* < 0.05

The analysis includes boys and girls separately

Using 6-year-old children as a reference, risk estimate for overweight and obesity was not significantly elevated in 7-, and 8-year-old girls but the risk was 1.5-times increased for overweight for 8-year old boys (*p* = 0.021). Regional differences were more dominant in obesity among boys than in girls. We detected a significantly higher risk (from 2.0 to 3.4 OR) in other regions for obesity compared to Central Hungary among boys. The effect of the level of urbanization was more characteristic in girls than in boys. Being obese has 2-times higher likelihood in rural girls compared to their urban counterparts (*p* = 0.003).

## Discussion

In the current study, the prevalence of overweight was 12.6% and obesity was found to be 8.6% in Hungarian 6–8-year-old children according to the IOTF criteria. Using the WHO growth reference, prevalence was slightly higher, 14.2% for overweight and 12.7% for obesity. Substantial regional differences were found in obesity rates with two-times higher prevalence in the region with the highest compared to the region with the lowest values. Besides, we observed significant sex differences in these varieties.. In our study, the urbanization level of settlements affected only obesity in girls, while regional location was relevant only for obesity in boys.

The prevalence of overweight and obesity among Hungarian children was recently presented in the IDEFICS study [32]. This study, which was conducted in eight European countries, described that 16.6% of boys and 18.2% of girls among 2–9.9 years-olds are overweight or obese in Hungary. A detailed comparison of our data with this study is limited because of the different age categorization of the children.

This paper is unique because this was the first time that the prevalence of overweight and obesity among children was assessed according to the geographical regions of Hungary in a nationally representative sample. The only study that assessed deprived and non-deprived regional differences in childhood obesity prevalence in Hungary was carried out by Bodzsar et al. [33] between 2010 and 2012 which compared the nutritional status among 3–18 years old children in deprived and non-deprived regions. The results of this study can be compared to our data. Regions were graded into ‘deprived’ and ‘non-deprived’ areas based on economic and social welfare indicators. Nutritional status was assessed by BMI using the IOTF criteria. Surprisingly, the prevalence of overweight and obesity did not differ between the deprived regions (girls: 19.8%, boys: 20.2%) and the national references (girls: 19.1%, boys: 21.5%), however they found difference in underweight. The authors did not explain this finding.

Interestingly, geographic distribution of obesity shown here was similar to the pattern observed earlier for obesity among Hungarian adults [34]. The prevalence rates both in adults and in children were the lowest in Central Hungary (26.8% (95% CI: 21.5, 33.0) for adults) and in Western Transdanubia (24.4% (95% CI: 15.0, 37.2) for adults). The prevalence of adult obesity was the highest in the Northern Great Plain (41.1% (95% CI: 31.04, 51.9)) and in Northern Hungary (33.6% (95% CI: 23.6, 45.3)). Obesity rate in our study was also high in Northern Hungary. The highest prevalence of childhood obesity, however, was found in Southern Transdanubia, but it did not appear to be high in adults. Many parents believe that obesity is an inherited problem, a genetic factor, which causes the excess weight gain, and do not consider how their own eating habits and the surrounding environment affect the lifestyle and, consequently, the weight status of their child [35]. Although, genetic predisposition for obesity can certainly play a role, but the rapidly rising prevalence in childhood obesity suggests that other factors (e.g. intake of energy-dense foods that are high in sugar and/or fat, sedentary lifestyle, transportation, urbanization, low rate of breastfeeding, food processing, aggressive marketing to children etc.) contribute more significantly to this problem [11, 36–39].

To explain the observed regional differences, we examined GDP per region. There is a growing evidence about an inverse association between GDP and the prevalence of overweight and obesity at country level [38]. In line with this, we observed the lowest prevalence of overweight and obesity in the region where GDP was the highest (19,532.7 USD per capita) and the highest prevalence rates in the area where GDP was one of the lowest (8286.8 USD per capita) [40]. Egger et al. have found similar results based on data coming from 175 countries [41]. In this work GDP has been significantly associated with adult BMI. We know that economic growth, nutrition habits and environmental characteristics are interlinked [42]. For instance, population living in developed countries are more likely to be exposed to an obesogenic environment which usually leads to overconsumption. Consumption driven increases in GDP may be beneficial in the developing economies, but the detrimental impacts of the over-consumption they have created in wealthy countries are now becoming apparent. Another explanation could be behind this phenomenon that higher income seems to be related to healthier dietary patterns [43] in the developed countries.

Regional differences have been described in several other COSI countries: in Italy, Portugal, Serbia, Sweden, Malta, and Greece [5–8, 44–46]. The prevalence of obesity was twice as high in southern than in northern Italy. Interestingly, similar geographic gradient can be seen for a wide variety of pediatric health indicators such as education level, poverty or access to and efficiency of health services [44]. A Portuguese study showed a higher risk of obesity in the islands region. It has been linked to a range of factors, including low levels of physical activity and a decline in the consumption of the traditional foods of the islands, such as fresh fish, meat, and local fruits and vegetables, which have been replaced with a high-energy-dense diet [7]. A study from Sweden also adds to existing evidence of a persisting north-south gradient in childhood obesity across Europe [5]. An examination described similar results in Serbia as in Italy and Sweden. Children from the northern part of the country were less likely to be overweight and obese than children from the south-central region of Serbia. Overweight and obesity were strongly associated with poor local community development and lower level of urbanization [6]. In Greece, there was a parallelism between regional differences and urbanization levels. Greece reported a higher risk of becoming obese for children in urban environments which might be due to differences in lifestyle and socioeconomic factors. The abrupt urbanization in Greece might have resulted in worsening living conditions in families moving to bigger cities from villages [46].

Concerning the impact of level of urbanization on overweight and obesity, we found higher prevalence rates in rural than in urban areas, although results were significant only for obesity in girls. Our findings are similar to that of other European countries, like Norway, Iceland, Sweden and Serbia [6, 47–49]. The prevalence of overweight and obesity is reverse in Portugal and Turkey in rural/urban areas [7, 8]. We found more remarkable differences between the seven geographical regions than between urban, semi-urban and rural areas.

The multivariable analysis showed that, after controlling for the independent variables, few factors remained significant predictors of overweight and obesity, and that the relevance of factors differed by sex. Similar to our findings, the multivariable analysis was conducted separately by sex in the Swedish COSI study, where notable differences were described between boys and girls [5]. Concerning obesity, data from boys showed increased risk in rural and semi-urban areas compared to urban areas. In contrast, no urban-rural gradient was found in the prevalence of obesity in the data for girls. Authors described that parents are less likely to encourage sons to lose weight, perhaps because the ideal male body shape is more muscular [50]. Besides, maternal restriction of snacks is more common in case of daughters [51]. Further reasons for this sex-effect on the differences in obesity prevalence is currently not well studied thus further research is needed to confirm the results and understand the underlying causes [52, 53].

This study has a number of strengths and limitations. Strengths of our study include the large sample size, which is representative of 6–8-year-old children in the total population as well as the standardized weight and height measurements and the application of a consistent data collection protocol. A further strength is that using two different criteria enables other countries to make multiple comparisons. All measurements were conducted by trained personnel according to detailed standard operating procedures. Strengths include also that our study described the prevalence of overweight and obesity according to the seven geographical regions the first time. A limitation of the study is that we have no information about the SES status on individual level (e.g. parental education, family income) which could have helped us to deeper understand the observed differences.

## Conclusions

Overweight and obesity are emerging problems in Hungary. There are remarkable differences in the prevalence of obesity by sex between geographic regions. Policymakers and experts should design and implement targeted strategies to reduce regional inequalities. Besides, sex-specific varieties in obesity should be considered when an intervention is developed e.g. more effective parental education is needed for families with overweight male children. Finally, further research is needed to confirm our results and, particularly, to expand the knowledge and understand the causes behind observed sex differences.

## Abbreviations

BMI: Body Mass Index
COSI: Childhood Obesity Surveillance Initiative
GDP: Gross Domestic Product
IOTF: International Obesity Task Force
SES: Socioeconomics status
WHO: World Health Organization
